# Prediction of Driver Modules via Balancing Exclusive Coverages of Mutations in Cancer Samples

**DOI:** 10.1002/advs.201801384

**Published:** 2018-12-18

**Authors:** Bo Gao, Yue Zhao, Yang Li, Juntao Liu, Lushan Wang, Guojun Li, Zhengchang Su

**Affiliations:** ^1^ School of Mathematics Shandong University Jinan 250100 China; ^2^ State Key Laboratory of Microbial Technology Shandong University Jinan 250100 China; ^3^ IAM MADIS NCMIS Academy of Mathematics and Systems Science Chinese Academy of Sciences Beijing 100190 China; ^4^ School of Mathematical Sciences University of Chinese Academy of Sciences Beijing 100049 China; ^5^ Department of Bioinformatics and Genomics College of Computing and Informatics The University of North Carolina at Charlotte 9201 University City Blvd Charlotte NC 28223 USA

**Keywords:** cancer genomics, coverage, driver modules, exclusivity, signaling networks

## Abstract

Mutual exclusivity of cancer driving mutations is a frequently observed phenomenon in the mutational landscape of cancer. The long tail of rare mutations complicates the discovery of mutually exclusive driver modules. The existing methods usually suffer from the problem that only few genes in some identified modules cover most of the cancer samples. To overcome this hurdle, an efficient method UniCovEx is presented via identifying mutually exclusive driver modules of balanced exclusive coverages. UniCovEx first searches for candidate driver modules with a strong topological relationship in signaling networks using a greedy strategy. It then evaluates the candidate modules by considering their coverage, exclusivity, and balance of coverage, using a novel metric termed exclusive entropy of modules, which measures how balanced the modules are. Finally, UniCovEx predicts sample‐specific driver modules by solving a minimum set cover problem using a greedy strategy. When tested on 12 The Cancer Genome Atlas datasets of different cancer types, UniCovEx shows a significant superiority over the previous methods. The software is available at: https://sourceforge.net/projects/cancer‐pathway/files/.

## 1 Introduction

Cancer is a complex genetic disease associated with aberrant genomic alterations in germline or somatic genomes. Large‐scale cancer genome sequencing projects, such as The Cancer Genome Atlas (TCGA),^1^ the International Cancer Genome Consortium (ICGC),^2^ and the Cancer Cell Line Encyclopedia (CCLE),^3^ have systematically profiled genomic alterations for various cancer types. A key challenge for these projects is to distinguish cancer‐causing driver mutations from inconsequential passenger mutations.^4^, ^5^ Despite significant progress of mutation‐based cancer gene prediction, the long tail phenomenon, i.e., there are much more infrequently mutated genes than frequently mutated ones in cancer genomes, complicates the efforts to identify infrequently mutated driver genes.^6^, ^7^

A common hypothesis is that cancer is a disease of pathway defects caused by mutations in genes involved.^8^, ^9^ An increasing body of evidence suggest that driver mutations tend to fall into a limited number of cellular signaling and regulatory pathways controlling cell proliferation.^9^, ^10^, ^11^ The observed mutational heterogeneity in cancer can be explained by different combinations of driver mutations in cancer cells. Several methods have been developed to identify significantly mutated known pathways or networks. Clearly, these methods can be limited by the currently incomplete known pathways and interaction networks in databases.^12^, ^13^

De novo computational methods have been developed to discover driver pathways based on two observations: 1) a large number of cancer samples have mutated genes in each driver pathway or module, referred to as high coverage property, where the coverage of a gene or a module is defined as the number of samples in which the gene or genes in the module are mutated; and 2) genes in a single driver pathway are altered exclusively in a cancer sample, referred to as high exclusivity,^14^, ^15^, ^16^, ^17^ i.e., at most one driver gene in a driver pathway is mutated in most samples. The phenomenon has led to the hypothesis that mutation in a single driver gene is usually sufficient to disturb the pathway and drive the cancer.

**Figure 1:**
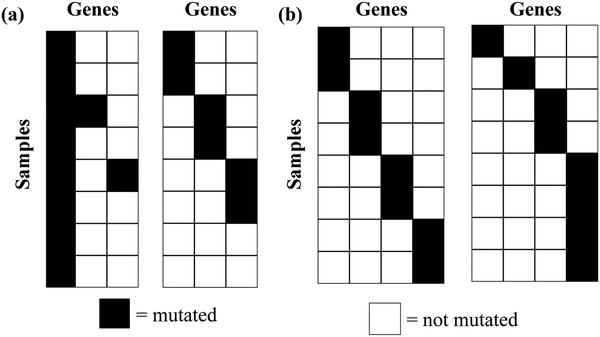
a) Two mutation matrices *M*_1_ (left) and *M*_2_ (right) with different coverage and exclusivity can have the same Dendrix function value *W*(*M*_1_) = *W*(*M*_2_) = 6. In *M*_1,_ the function value *W*(*M*_1_) is dominated by the gene with the highest mutation frequency, and the genes are not exclusive, but in *M*_2,_ genes are strictly exclusive and the coverages of the genes are balanced. b) Two strictly exclusive matrices *M*_3_ (left) and *M*_4_ (right) with the same coverage can exhibit different coverage patterns which can be evaluated by different entropy scores. The coverage of *M*_3_ is evenly contributed by the genes with similar mutation frequencies with a larger entropy score = 2.0, while the coverage of *M*_4_ is unbalanced with a smaller entropy score = 1.75.

Miller et al.^18^ proposed the RME algorithm for identifying gene modules using an online‐learning method considering pairwise exclusivity. Then Vandin et al.^19^ designed Dendrix based on a function that balances coverage and mutual exclusivity of a gene module, and was later optimized by different authors.^19^, ^20^, ^21^, ^22^ Subsequently, Leiserson et al.^21^ and Zhang et al.^23^ developed Multi‐Dendrix and CoMDP, respectively, to generalize Dendrix, so that it could simultaneously measure coverage and exclusivity of multiple gene modules. Zhang et al.^24^, ^25^ further generalized Dendrix to study common and specific cancer driver gene sets. However, due to the long tail phenomenon in cancer mutation profiles, genes with high mutation frequencies may dominate the Dendrix‐based function in real application (**Figure** **1**a),^26^, ^27^ thus missing less frequently mutated driver genes.

Methods that integrate other biological information have also been proposed to identify driver modules, such as protein‐protein interaction (PPI) networks and gene expression data. For example, HotNet^28^ and HotNet2^29^ identify mutated subnetworks with large coverage. DriverNet^30^ predicts driver genes by integrating both PPI networks and expression data. MEMo^31^ and MEMCover^32^ can identify exclusive modules based on PPI networks, but they suffer from high computational complexity.^29^, ^31^ To reduce the computational cost, Kim et al.^33^ and Leiserson et al.^34^ designed WeSME and WExT, respectively.

More recently, efforts have been made to address the challenge of identifying mutual exclusive modules. For example, Szczurek et al.^35^ proposed muex based on a probabilistic generative model, while Babur et al. designed mutex^36^ to score exclusivity of a gene module by testing the significance of the mutation profile of each gene in the module against the union of all other genes in the module. In our earlier method CovEx,^27^ we defined an exclusive fraction for each gene in a module, and further defined the exclusivity of the gene module as the average exclusive fraction over the genes in the module. Both the mutex and CovEx scores account for the degree at which each gene in a module contributes to the exclusive score of the module. However, the gene modules identified by these existing methods may exhibit different exclusive properties (Figure 1b) due to the heterogeneity and the long tail of cancer mutation profiles. Especially, the coverage of some identified exclusive gene modules may be still dominated by few genes, leading to false predictions.

**Figure 2:**
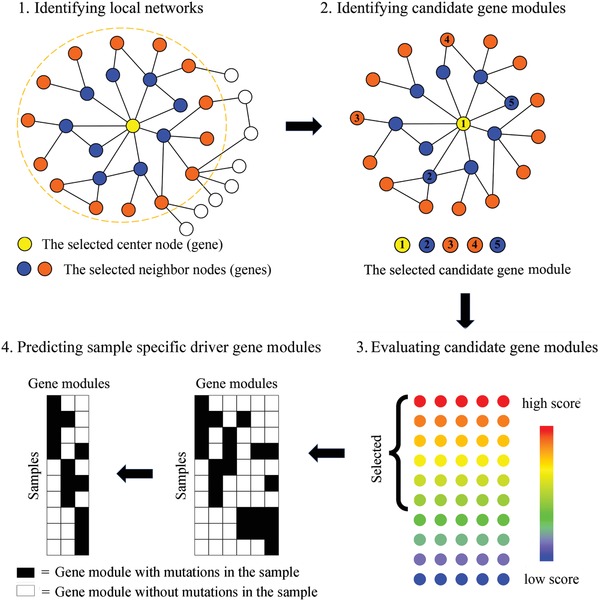
Flowchart of the UniCovEx algorithm. 1) We search for the local networks for the considered genes in the influence network derived from a PPI network. 2) For each local network, we identify candidate gene modules using an exclusive greedy strategy. 3) We evaluate each candidate gene module and select the ones with high scores. 4) We predict the sample‐specific driver modules and the pathways by modeling the task as a minimum set cover problem.

To circumvent the obstacle, Leiserson et al.^26^ developed CoMEt emphasizing on balanced mutual exclusivity using an exact statistical test. A module is said to be balanced exclusive if all genes in the module have almost the same mutation frequency in a set of cancer samples that have only one mutated gene in the module. In fact, an exclusive driver module of unbalanced exclusive coverage would be viewed as a combination of several modules of nearly balanced exclusive coverage. Study on gene modules with balanced exclusive coverages will enable the discovery of combinations of low frequency alterations that cannot be recognized by previous methods. However, CoMEt suffers from high computational complexity of the proposed metric evaluating the balance of mutual exclusivity, and thus limits its applications. To overcome this limitation and also to further improve the accuracy of driver module identification, we have developed UniCovEx. To this end, we introduced a new metric called exclusive entropy to measure the degree at which an exclusive module is balanced. The bigger the exclusive entropy of a module, the more balanced the exclusive module (Experimental Section). UniCovEx starts by finding local networks in a large influence network obtained by a random walk model on a PPI network (step 1 in **Figure** **2**). Then UniCovEx identifies candidate modules in each local network (step 2 in Figure 2), and evaluates each of them using a score function that integrates the coverage, mutual exclusivity, and degree of balance of the candidates (step 3 in Figure 2). Finally, UniCovEx predicts the patient/sample‐specific driver modules in each cancer sample (step 4 in Figure 2). When evaluated on 12 cancer types annotated by the TCGA project, UniCovEx outperformed previous methods in identifying known driver modules in most cancer types. Enrichment analyses indicate that UniCovEx may also predict novel driver genes and modules. UniCovEx is also more computationally efficient than existing methods due to the greedy strategies used.

## 2 Results

### 2.1 UniCovEx Outperforms State‐of‐the‐Art Methods in Identifying Known Cancer Driver Modules

To evaluate the performance of our method, we compared it with two state‐of‐the‐art methods CovEx^27^ and HotNet2^29^ on 3106 samples for 12 cancer types (Table S1, Supporting Information) using 1571 cancer genes documented in the NCG 5.0 repository^37^ and three PPI networks, HINT+HI2012, iRefIndex, and Multinet. We consider all samples of a cancer type as a dataset. We applied our method to each dataset/cancer type using the three PPI networks. For each dataset, we consider candidate modules that were identified by at least two PPI networks as the final predictions, and refer to them as consensus gene modules. To make the comparison fair, we extracted the consensus gene modules predicted by CovEx^27^ and HotNet2^29^ from the original publications for the same datasets using the same three PPI networks.

**Figure 3:**
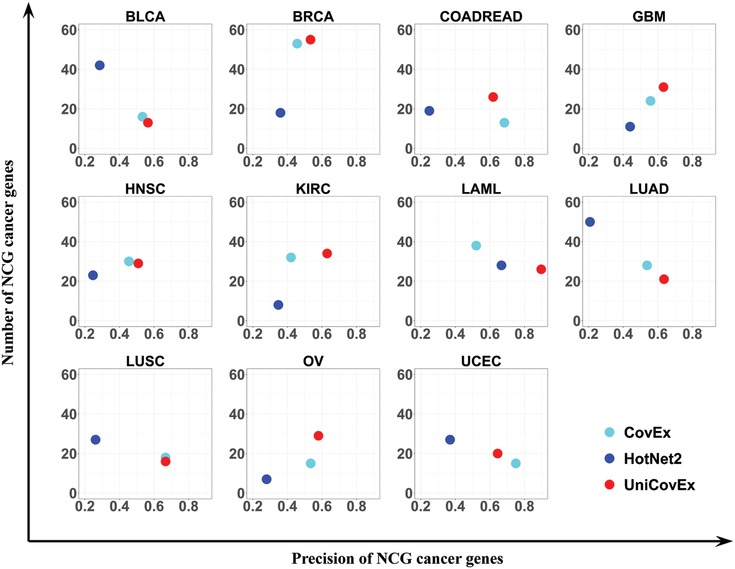
Precision and numbers of recovered NCG cancer genes by different methods for each cancer type.

As shown in **Figure** **3**, the precision of UniCovEx is higher than 50% (Table S2, Supporting Information) and also higher than that of HotNet2 and CovEx for all cancer types except colon adenocarcinoma and rectum adenocarcinoma (COADREAD) and uterine corpus endometrioid carcinoma (UCEC) for CovEx. In addition, the precision of CovEx is larger than that of HotNet2 for all the cancer types except acute myeloid leukemia (LAML). The numbers of NCG cancer genes identified by UniCovEx and CovEx are both larger than those of HotNet2 for six cancer types. UniCovEx generally identified more NCG cancer genes than CovEx.

**Figure 4:**
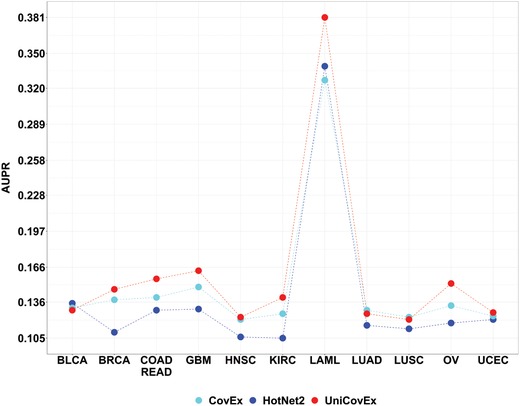
The AUPR values of different methods for each cancer type.

Clearly, in some datasets there is a tradeoff between the precision and the number of identified cancer genes. We plotted the precision‐recall (PR) curve by considering all the mutated genes, and calculated the values of area under the PR (AUPR) curve for each cancer type. As shown in **Figure** **4**, the AUPRs of UniCovEx are the largest for eight cancer types and the AUPRs of HotNet2 are the smallest for nine cancer types.

To more accurately compare the performance of different methods, we computed relative AUPR scores for UniCovEx and CovEx with respect to the AUPR of HotNet2 for each cancer type and thus the relative AUPR for HotNet2 is 1. Take breast invasive carcinoma (BRCA) as an example, we calculated the relative AUPRs of UniCovEx and CovEx to be 1.339 and 1262, respectively, indicating that the performance of UniCovEx and CovEx has been improved by 33.9% and 26.2%, respectively, relative to that of HotNet2, and that the performance of UniCovEx has been improved by 7.7% with respect to CovEx. As summarized in Table S3 in the Supporting Information, the performance of UniCovEx and CovEx has been improved by more than 10% for seven and five cancer types, respectively, in comparison with that of HotNet2. In addition, the performance of UniCovEx and CovEx has been improved by more than 20% for five and only one cancer types, respectively. The performance of UniCovEx has been even improved by more than 30% for BRCA and kidney renal clear cell carcinoma (KIRC).

Compared to CovEx, UniCovEx had better performance for eight cancer types and were more than 10% higher for five cancer types. Especially, the performance of UniCovEx has been improved by more than 15% for LAML and ovarian serous cystadenocarcinoma (OV) with respect to that of CovEx. Furthermore, the performance of UniCovEx was only 3% lower than that of CovEx for all three cancer types where CovEx performed better. Furthermore, CovEx obtains consensus gene modules based on 24 groups of results (eight for each PPI network) by solving eight linear programming problems for each considered gene, thus is computationally expensive. In contrast, UniCovEx finds consensus modules by searching only a single module for a gene using a greedy strategy. We tested the two methods on a computing server, and ran CovEx using the mathematical programming solver gurobi 7.5.2. As shown in Tables S4,S5 in the Supporting Information, UniCovEx completed the jobs in a few minutes while CovEx in hours. In addition to PR curves that give a rather informative picture of the methods' performance due to the highly skewed nature of the datasets, we also plotted the receiver operating characteristic (ROC) curves,^38^, ^39^, ^40^ and compared area under the ROC (AUC) curve values that also show the advantage of UniCovEx over the other methods (see Figure S1, Supporting Information).

### 2.2 UniCovEx as a Tool for Identifying Driver Genes and Modules in Cancers

The superior performance of UniCovEx positions it as a tool of choice to find driver genes and modules in cancer, thereby providing new insights into pathogenic mechanisms of cancers as well as new targets of therapies. Below we demonstrate the power of UniCovEx for such purposes using three cancer types as examples: acute myeloid leukemia (LAML), lung adenocarcinoma (LUAD), and lung squamous cell carcinoma (LUSC). We choose these three cancer types as they represent the full range of UniCovEx's performance: better in LAML and worse in LUAD and LUSC (Figure 4). However, UniCovEx still achieves rather high precision for both LUAD and LUSC.

#### 2.2.1. Acute Myeloid Leukemia (LAML)

**Figure 5:**
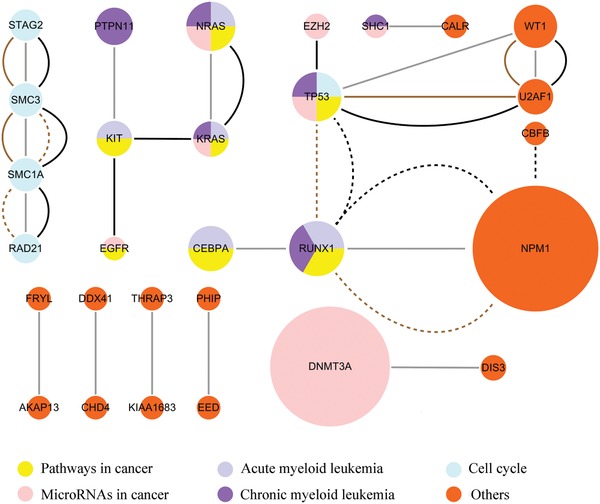
The LAML consensus gene modules identified by UniCovEx. The sizes of nodes (genes) are proportional to the mutation frequencies of the corresponding genes. Genes with the same/different color belong to the same/different KEGG pathways. Genes connected by different colored or dotted edges correspond to the identified gene modules of different sizes. The size of a gene module is the number of genes in the gene module.

We identified a total of 29 driver genes in 23 consensus gene modules (**Figure** **5**). Enrichment analysis against the genetic association database (GAD)^41^ using DAVID,^42^, ^43^ indicates that these genes were significantly enriched for “leukemia, myeloid, acute” (p = 3.72E‐15) and “leukemia, myelomonocytic, chronic” (p = 1.32E‐11). Especially, the identified genes NRAS, NPM1, RUNX1, WT1, and PTPN11 are related to both diseases, CEBPA, DNMT3A, TP53 and KIT are related to “leukemia, myeloid, acute” and KRAS is related to “leukemia, myelomonocytic, chronic”.

Enrichment analysis against the KEGG pathway database^44^ using DAVID^42^, ^43^ revealed that these genes were significantly enriched for chronic myeloid leukemia (hsa05220, p = 1.11E‐06), LAML (hsa05221, p = 1.37E‐05), central carbon metabolism in cancer (hsa05230, p = 2.34E‐05), microRNAs in cancer (hsa05206, p = 8.04E‐05), Ras signaling pathway (hsa04014, p = 2.86E‐04), cell cycle (hsa04110, p = 3.11E‐04) and pathways in cancer (hsa05200, p = 4.69E‐04). The identified genes NRAS, KRAS, TP53, SHC1, RUNX1, and PTPN11 were significantly enriched for chronic myeloid leukemia, and CEBPA, NRAS, KRAS, KIT, and RUNX1 were significantly enriched for LAML.

It should be noted that 26 out of the 29 genes predicted by UniCovEx are NCG cancer genes with exception for FRYL, KIAA1683, and SHC1. However, FRYL is an important gene in therapy‐related acute leukemias,^45^ while KIAA1683 and SHC1 might be novel cancer genes. In contrast, 38 out of 73 genes and 28 out of 42 genes identified by CovEx and HotNet2 are NCG cancer genes, respectively. These results further demonstrate the superiority of UniCovEx over the other algorithms to identify true cancer genes.

#### 2.2.2. Lung Adenocarcinoma (LUAD)

**Figure 6:**
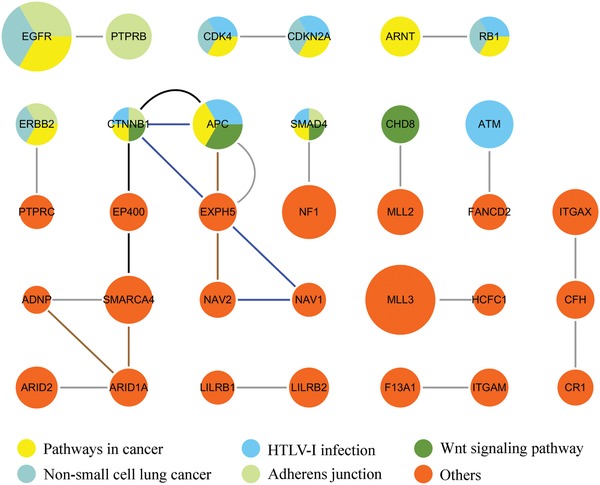
The LUAD consensus gene modules identified by UniCovEx. The meanings of the nodes and edges are the same as in Figure 5.

We identified a total of 33 driver genes in 19 consensus gene modules for LUAD (**Figure** **6**). The GAD enrichment analysis indicates that the genes were significantly enriched for pancreatic neoplasms (p = 3.07E‐09), epithelial ovarian cancer (p = 4.18E‐08), bladder cancer (p = 5.27E‐08), lung cancer (p = 5.75E‐07), and breast cancer (p = 5.94E‐07). Especially, the identified genes EGFR, CDKN2A, ERBB2, CFH, RB1, CDK4, ATM, ARNT, CTNNB1, and APC were enriched for lung cancer. The genes were also enriched for KEGG pathways such as pancreatic cancer (hsa05212, p = 2.33E‐06), bladder cancer (hsa05219, p = 1.04E‐05), pathways in cancer (hsa05200, p = 3.35E‐05), and non‐small cell lung cancer (hsa05223, p = 3.64E‐05). Especially, the identified genes EGFR, CDKN2A, ERBB2, RB1, and CDK4 were significantly enriched for non‐small cell lung cancer, demonstrating that UniCovEx accurately identified the cancer‐related gene modules.

Of the 33 genes identified for LUAD, 21 are NCG genes. Of the 12 genes (CFH, CHD8, CR1, EP400, F13A1, HCFC1, ITGAM, ITGAX, LILRB2, NAV1, and NAV2) that are not NCG cancer genes, several genes have been reported to be related to lung cancer. For instance, CFH has been known to inhibit the complement pathway and to contribute to tumor growth. CFH was also reported to be associated with lung cancer.^46^ CR1 plays a crucial role in carcinogenesis and has been proved to be associated with an increased risk of developing non‐small cell lung cancer in Chinese population.^47^ LILRB2 is also suggested to be important for lung cancer development.^48^ ITGAX has been confirmed to be a biomarker for noninvasive molecular diagnosis of lung cancer.^49^ In contrast, only 28 out of the 52 genes and 50 out of the 240 genes identified by CovEx and HotNet2 are NCG cancer genes, respectively. The precision of UniCovEx (63.6%) is also higher than that of CovEx (53.8%) and HotNet2 (20.8%).

#### 2.2.3. Lung Squamous Cell Carcinoma (LUSC)

**Figure 7:**
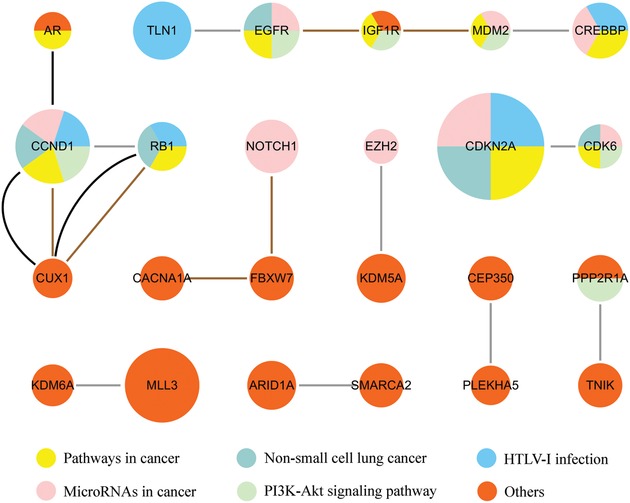
The LUSC consensus gene modules identified by UniCovEx. The meanings of the nodes and edges are the same as in Figure 5.

We identified a total of 24 genes in 13 consensus gene modules for LUSC (**Figure** **7**). The GAD enrichment analysis revealed that these genes were significantly enriched for breast cancer (p = 4.50E‐11), head and neck cancer (p = 2.17E‐07), esophageal cancer (p = 1.54E‐06), epithelial ovarian cancer (p = 4.82E‐06), and lung cancer (p = 7.47E‐06). Eight genes including EGFR, IGF1R, FBXW7, CCND1, CDKN2A, EZH2, MDM2, and RB1 were enriched for lung cancer. The genes were also enriched for KEGG pathways such as glioma (hsa05214, p = 4.07E‐09), melanoma (hsa05218, p = 7.01E‐09), and non‐small cell lung cancer (hsa05223, p = 6.55E‐06).

Of the 24 genes identified for LUSC, 16 genes are NCG genes. In contrast, 18 out of the 27 genes and 27 out of the 103 genes identified by CovEx and HotNet2 are NCG cancer genes, respectively. The precision of UniCovEx (66.7%) is the same as that of CovEx but is higher than that of HotNet2 (26.2%). Surprisingly, only eight identified genes (CCND1, CDK6, CDKN2A, KDM5A, MDM2, MLL3, PPP2R1A, and RB1) are shared by UniCovEx and CovEx, and they are all NCG cancer genes. Thus, the predicted gene modules for LUSC by UniCovEx are quite different from the other methods. We also compared the genes identified for LUAD and LUSC and found only five genes (ARID1A, CDKN2A, EGFR, MLL3, and RB1) are common for the two cancer types, demonstrating their different disease mechanism.

## 3 Discussion

Identifying high coverage and mutually exclusive gene modules is of great significance to characterize the driver pathways. However, the existing methods are usually affected by the long tail of cancer mutation profiles that mutation frequencies of a large number of genes are very small. Different identified exclusive gene modules may exhibit different exclusive coverages. To tackle this problem, we designed the exclusive entropy score to evaluate the exclusive coverage of gene modules. By integrating the exclusive entropy score with the exclusive score of gene modules defined in CovEx, we designed a comprehensive score considering coverage, mutual exclusivity, and exclusive coverage simultaneously for evaluating a candidate gene module.

We present an efficient method UniCovEx for identification of mutually exclusive gene modules with balanced exclusive coverages. Generally, the topologically related genes in PPI networks are more likely to be functionally related and correspond to real pathways and functional modules. UniCovEx first identifies candidate gene modules by a greedy approach in each of the local network of an influence network. The influence network evaluates the topological relationships of genes in the PPI network. Then the identified gene modules are evaluated by their UniCovEx scores and the high scoring modules are selected. We further predict sample‐specific driver modules by solving a minimum set cover problem using a greedy method.

We consider modules output by at least two of the three PPI networks (HINT+HI2012, iRefIndex, and Multinet) as the final predictions. Applied to 12 cancer types, UniCovEx identified numerous cancer‐related gene modules, and largely outperformed two state‐of‐the‐art methods. The enrichment analysis of complex disease and KEGG pathways reveals the possible driver genes and pathways for each cancer type. Compared to the previous methods, UniCovEx shows significant advantage for single cancer type analysis.

The limitation of UniCovEx is that it tends to identify strictly exclusive gene modules in practice and ignore meaningful gene modules with relatively poor exclusivity. New candidate module identification methods with better balance between coverage and exclusivity may further improve the performance of UniCovEx.

## 4 Conclusions

We present a new method UniCovEx for identifying topologically related gene modules with balanced exclusive coverages. The minimum set cover model is applied to predict sample‐specific driver gene modules based on the mutation profile of sample populations. Applied to multiple cancer types, UniCovEx identifies a number of cancer driver gene modules and shows significant advantage over other methods for multiple cancer types. The identified gene modules are enriched for many known cancer pathways. UniCovEx can be very useful for understanding the pathologic mechanisms, target identification, diagnosis, and personalized treatment of cancer.

## 5 Experimental Section

*Datasets*: The TCGA cancer datasets that were analyzed using HotNet2 were downloaded.^29^ The datasets contained 3106 samples for 12 cancer types including bladder urothelial carcinoma (BLCA), breast invasive carcinoma (BRCA), colon adenocarcinoma (COAD), glioblastoma multiforme (GBM), head and neck squamous cell carcinoma (HNSC), kidney renal clear cell carcinoma (KIRC), acute myeloid leukemia (LAML), lung adenocarcinoma (LUAD), lung squamous cell carcinoma (LUSC), ovarian serous cystadenocarcinoma (OV), rectum adenocarcinoma (READ), and uterine corpus endometrioid carcinoma (UCEC). In the analysis, COAD and READ were combined into one type, denoted as COADREAD. A total of 11565 genes were documented after filtration with RNA‐seq expression data. The influence matrix files were downloaded for three PPI networks HINT+HI2012, iRefIndex, and Multinet from http://compbio‐research.cs.brown.edu/pancancer/hotnet2/, which were generated using a random walk model.^29^

*Identification of Candidate Gene Modules*: The weighted complete influence graph was obtained from HotNet2,^29^ which was derived from a PPI network using an insulated heat diffusion process model. A reduced influence network was constructed by removing small weight edges^27^ on the network, so that the resulting network had an average degree of 15, which was close to the average degree of a real PPI network. The edges in the reduced influence network reflected strong topological relationships between the corresponding gene pairs in a better way.

Local networks were extracted by breadth‐first search on the reduced influence network, starting at a node and gradually exploring its neighbor nodes until reaching a maximal number of genes (100 in the experiments). Candidate modules in each resulting local network were searched for using an iterative greedy method. The center node of the local network was first added to the originally empty candidate module, and then in each subsequent iteration, a gene that maximizes its exclusive score was added to the module. If multiple genes contributed to the same exclusive score, the gene that contributes to the largest coverage of the candidate gene module was added. The iteration ended when the resulting candidate module reached the maximum size (five in the experiments) or the exclusive score of the resulting candidate module was less than a cutoff (0.95 in the experiments). The greedy method only considered genes mutated in at least two samples in the experiments.

*Evaluation of Candidate Gene Modules*: Based on the exclusive score introduced in CovEx,^27^ the exclusive entropy of a gene module was introduced. For a gene module *M =* {*g*_1_, *g*_2_, …, *g_s_*}, the sample set in which *g_i_* was mutated was denoted by *P_i_*, and the sample set in which *g_i_* was exclusively mutated was denoted by EP*_i_*, for *i* = 1, 2, …, *s*. Let *n_i_*_=_ |*P_i_*| and *n*′*_i_* = |*EP_i_*|. *P_i_* was called mutated samples and *EP_i_* exclusively mutated samples for *g_i_*. Then the exclusive degree of gene *g_i_* was denoted by Ex(*g_i_*), i.e., Ex(*g_i_*) = *n*′*_i_/n_i_*, and the exclusive score of the gene module *M* was denoted by Ex(*M*), i.e., $\mathrm{Ex}(M)=\sum_{i=1}^{i=s}\mathrm{Ex}(g_{i})/s$. To quantify the degree at which genes in module *M* were evenly mutated in the samples and therefore balanced, the mutation ratio *p_i_* = *n*′*_i_*/*n*′ was calculated for each gene *i*, where *n*′ is the number of all exclusively mutated samples for all the genes in module *M*. The exclusive entropy score $H(M)=-\sum_{i=1}^{i=s}p_{i}\log_{2}p_{i}$ was then calculated. Finally, each module *M* was evaluated using a score defined as UniCovEx(*M*) = cov(*M*)Ex(*M*)*H*(*M*), where cov(*M*) is the minimum coverage for the genes in *M*. Therefore, the UniCovEx score integrates coverage, mutual exclusivity, and balanced degree of exclusive coverage.

Each candidate module was evaluated using its UniCovEx score. For each candidate gene module, the sub‐modules of smaller sizes identified in the iterations of the greedy method were also considered. For candidate modules with the same size, the ones with larger UniCovEx scores were selected orderly until at least one gene in a module was mutated in each sample, and thus each sample was covered.

*Prediction of Sample‐Specific Driver Gene Modules*: It was intended to predict the driver gene modules for a specific cancer sample by finding the minimum number of gene modules from the selected high scoring modules such that each sample has at least one mutated gene in the union of the modules. This problem was modeled as the minimum set cover problem, which unfortunately is NP hard, however. Therefore, an iterative greedy strategy was resorted to. In each iteration, a gene module was selected such that the number of covered samples was maximized. Due to the bias of this strategy to large‐sized modules, modules of different sizes were considered separately. In this study, candidate gene modules with a size ranging from 2 to 5 genes, resulting in four groups of sample‐specific gene modules with different sizes for each PPI network were considered.

*Evaluation of the Performance of Methods*: The prediction precision of an algorithm on a dataset/cancer type was defined as the fraction of identified NCG cancer genes to the union of the genes in the consensus modules output by the algorithm. The recall was defined as the fraction of identified NCG cancer genes to all the NCG genes mutated in the dataset/cancer type.

## Conflict of Interest

The authors declare no conflict of interest.

## Supporting information

Supplementary
