# Supplementary material for: Prediction of Driver Modules via Balancing Exclusive Coverages of Mutations in Cancer Samples
Source: Adv Sci (Weinh). 2018 Dec 18;6(4):1801384. doi: 10.1002/advs.201801384 (PMC6382311; doi:10.1002/advs.201801384)
Supplement: Supplementary file 1 — Supplementary [file ADVS-6-1801384-s001.pdf]

Copyright WILEY-VCH Verlag GmbH & Co. KGaA, 69469 Weinheim, Germany, 2018.

ADVANCED SCIENCE Open Access

Supporting Information

for *Adv. Sci.,* DOI: 10.1002/advs.201801384

Prediction of Driver Modules via Balancing Exclusive Coverages of Mutations in Cancer Samples

Bo Gao, Yue Zhao, Yang Li, Juntao Liu, Lushan Wang, Guojun Li, and Zhengchang Su**

Supplemental Material

Table of Content

Supplementary Tables
Supplementary Figures

Supplementary Tables

Table S1. The number of samples for each cancer type in the analysis.

Cancer type	Number of samples
BLCA	87
BRCA	763
COADREAD	193
GBM	290
HNSC	293
KIRC	417
LAML	192
LUAD	185
LUSC	160
OV	316
UCEC	210

Table S2. The numbers of UniCovEx output genes and NCG genes, and precision of NCG genes for different cancer types.

Cancer type	Number of output genes	Number of NCG genes	Precision of NCG genes
BLCA	23	13	56.5%
BRCA	103	55	53.4%
COADREAD	42	26	61.9%
GBM	49	31	63.3%
HNSC	57	29	50.9%
KIRC	54	34	63.0%
LAML	29	26	89.7%
LUAD	33	21	63.6%
LUSC	24	16	66.7%
OV	50	29	58.0%
UCEC	31	20	64.5%

Table S3. The AUPRs and relative AUPRs for different methods in different cancer types.

Cancer type	Method	AUPR	relative AUPR
BLCA	UniCovEx	0.1287169	0.953922
	CovEx	0.1313144	0.973172
	HotNet2	0.1349344	1
BRCA	UniCovEx	0.1468897	1.33871
	CovEx	0.1384988	1.26224
	HotNet2	0.109725	1
COADREAD	UniCovEx	0.1559957	1.21388
	CovEx	0.1402761	1.09155
	HotNet2	0.1285104	1
GBM	UniCovEx	0.1628815	1.25402
	CovEx	0.1494833	1.15087
	HotNet2	0.1298877	1
HNSC	UniCovEx	0.1228755	1.15457
	CovEx	0.1209371	1.13635
	HotNet2	0.1064255	1
KIRC	UniCovEx	0.1400135	1.33009
	CovEx	0.1255438	1.19263
	HotNet2	0.1052661	1
LAML	UniCovEx	0.3809044	1.12338
	CovEx	0.3269303	0.964197
	HotNet2	0.3390701	1
LUAD	UniCovEx	0.1262391	1.08783
	CovEx	0.129308	1.11428
	HotNet2	0.1160464	1
LUSC	UniCovEx	0.1206289	1.06565
	CovEx	0.122941	1.08608
	HotNet2	0.1131973	1
OV	UniCovEx	0.1516513	1.28096
	CovEx	0.1330959	1.12423
	HotNet2	0.1183889	1
UCEC	UniCovEx	0.1272981	1.04969
	CovEx	0.1239583	1.02215
	HotNet2	0.1212719	1

Table S4. The CPU time of CovEx for each cancer type. For each PPI network, CovEx was run for both $\lambda=0$ and $\lambda=1$ with other default parameters.

Cancer type	HINT+HI2012	iRefIndex	Multinet
BLCA	4h 19m 3s	5h 0m 28s	4h 36m 41s
BRCA	5h 9m 33s	4h 58m 5s	4h 42m 2s
COADREAD	4h 25m 20s	4h 39m 58s	4h 26m 42s
GBM	4h 6m 16s	4h 25m 33s	4h 5m 18s
HNSC	6h 4m 26s	5h 45m 24s	5h 22m 42s
KIRC	4h 54m 42s	4h 37m 34s	3h 2m 22s
LAML	20m 57s	31m 33s	32m 44s
LUAD	3h 49m 3s	4h 6m 24s	5h 13m 48s
LUSC	6h 6m 13s	5h 51m 37s	5h 19m 0s
OV	4h 41m 31s	4h 49m 2s	3h 27m 7s
UCEC	4h 37m 49s	4h 53m 2s	4h 23m 58s

Table S5. The CPU time of UniCovEx for each cancer type. For each PPI network, UniCovEx was run with default parameters.

Cancer type	HINT+HI2012	iRefIndex	Multinet
BLCA	35s	56s	1m 13s
BRCA	1m 7s	1m 27s	1m 41s
COADREAD	34s	55s	1m 11s
GBM	35s	56s	1m 14s
HNSC	48s	1m 14s	1m 27s
KIRC	46s	1m 7s	1m 23s
LAML	28s	46s	1m 4s
LUAD	42s	1m 4s	1m 22s
LUSC	43s	1m 9s	1m 27s
OV	37s	58s	1m 16s
UCEC	40s	1m 0s	1m 19s

Supplementary Figures

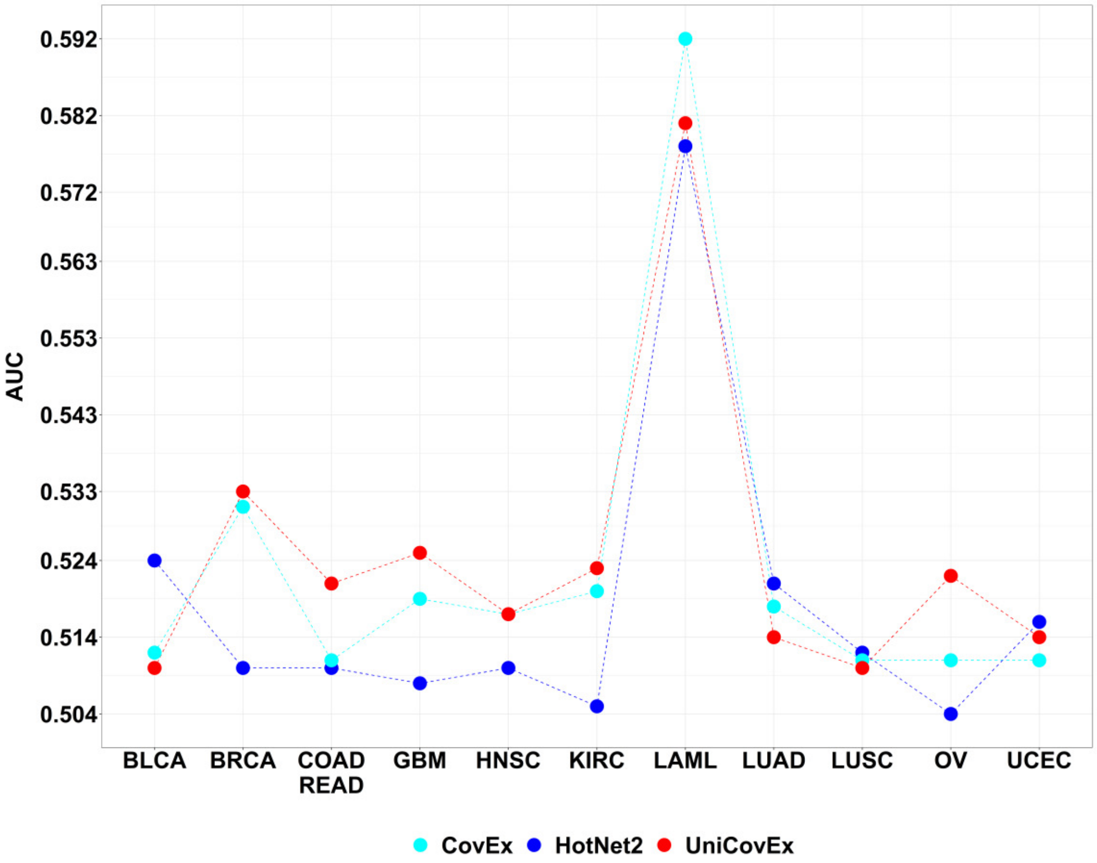

Figure S1. The AUC values of different methods for each cancer type.
